# Compatibility of endoclips in the gastrointestinal tract with magnetic resonance imaging

**DOI:** 10.1038/s41598-020-73726-5

**Published:** 2020-10-06

**Authors:** Dong Yeol Shin, Sumi Park, Ain Kim, Eung-Sam Kim, Han Ho Jeon

**Affiliations:** 1 Division of Gastroenterology, Department of Internal Medicine, National Health Insurance Service Ilsan Hospital, 100 Ilsan-ro Ilsan-donggu, Goyang-si, Goyang, 10444 Korea; 2 Department of Radiology, National Health Insurance Service Ilsan Hospital, Goyang, Korea; 3 Department of Human Biology, University of Toronto, Toronto, ON Canada; 4 Department of Biological Sciences and Research Center of Ecomimetics, Chonnam National University, Gwangju, Korea

**Keywords:** Gastroenterology, Risk factors, Materials science

## Abstract

There are no clear guidelines on the compatibility between endoclips that remain in the gastrointestinal (GI) tract and magnetic resonance imaging (MRI). The purpose of this study was to investigate the effect of 3T (T) MRI on endoclips placed in excised pig tissues. Two types of endoclips were assessed: Olympus EZ (HX-610-135L) and QuickClip Pro (HZ-202LR). We assessed tissue damage or perforation and detachment of endoclips under 3T MRI magnetic field. We also evaluated the magnitude of force required to detach the endoclips from the porcine tissue. We measured the magnetic force acting on the Olympus EZ clips. QuickClip Pro clips were used as a control in this study. There was no tissue damage and no detachment of the endoclips (Olympus EZ and QuickClip Pro) during 3T MRI. The force required to detach the Olympus EZ clips ranged from 0.9 to 3.0 N. The translational magnetic force acting on the endoclips was 3.18 × 10^–3^ N. Ex vivo experiments showed that the magnetic field generated by 3T MRI did not cause tissue damage or perforation and did not detach the endoclips. Olympus EZ clips and QuickClip Pro clips in the GI tract appear to be safe during 3T MRI.

## Introduction

Endoclips are metallic clips used for hemostasis, anchoring stents, closing intraprocedural perforations, and marking tumors or other structures. During a study conducted in 2009 by Gill et al., a 1.5T (T) magnetic field was applied to three types of endoclips (Resolution Clip, TriClip, and QuickClip) bound to a piece of gastric mucosa excised from a pig^1^. The TriClip (Cook Endoscopy, Winston-Salem, NC) detached while the other two endoclips remained attached to the gastric mucosa. In 2012, a patient whose esophageal bleeding was controlled using an endoclip known to be compatible with the magnetic resonance imaging (MRI), died of severe bleeding. It was suspected that bleeding might have been caused by clip migration during MRI^2^. Given the limited data on compatibility of certain endoclips with magnetic fields, MRI has been avoided or delayed for some patients.

Many types of stainless steel alloys and phases associated with different crystalline structures are currently used to manufacture endoclips. Their magnetic properties vary considerably, ranging from non-magnetic (austenitic grade) to highly magnetic (ferritic or martensitic grade). Both the magnetic field and friction between the clip and mucosal surface of the gastrointestinal (GI) tract are forces acting on endoclips during MRI.

In Korea, Olympus EZ clips are the most commonly used endoclips for GI tract procedures. However, the Olympus manual prohibits MRI on patients who have these clips within their GI tract due to the potential harm it can cause. Alternatively, QuickClip Pro clips enable patients to safely undergo an MRI after clip placement. This raises concerns about performing MRI on patients with endoclips. Thus, we aimed to determine the compatibility of commercially available Olympus GI clips with MRI. We evaluated tissue damage (including perforation) by endoclips or detachment of endoclips from the GI tissue under a standard 3T MRI magnetic field. Due to its safety during MRI, QuickClip Pro clips were used as controls in this study.

## Methods

**Figure 1:**
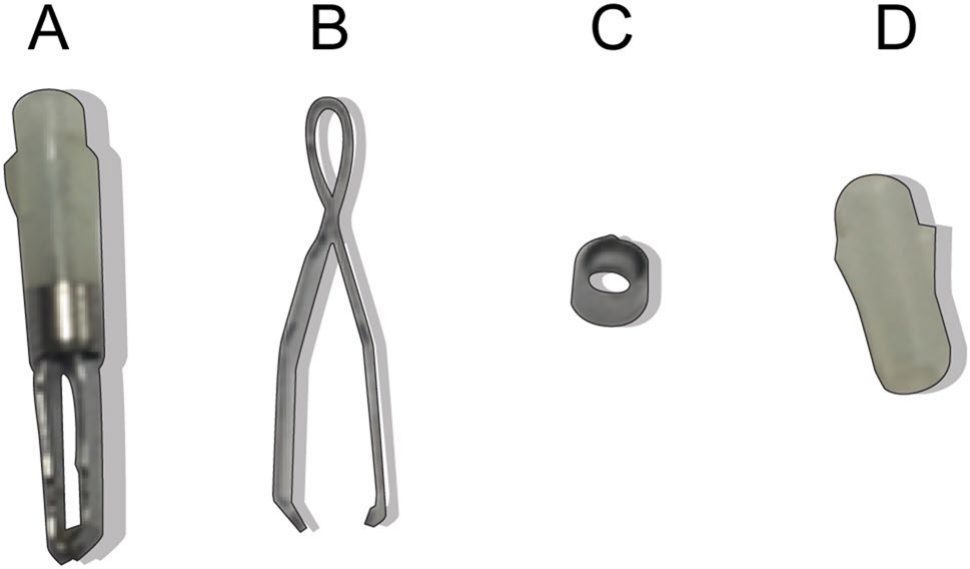
Endoclips used in the experiments, separated into metallic and non-metal components (**A**) Assembled Olympus EZ clip (HX-610-135L), (**B**) Metallic part 1 of HX-610-135L, (**C**) Metallic part 2 of HX-610-135L, (**D**) Non-metallic part of HX-610-135L.

**Figure 2:**
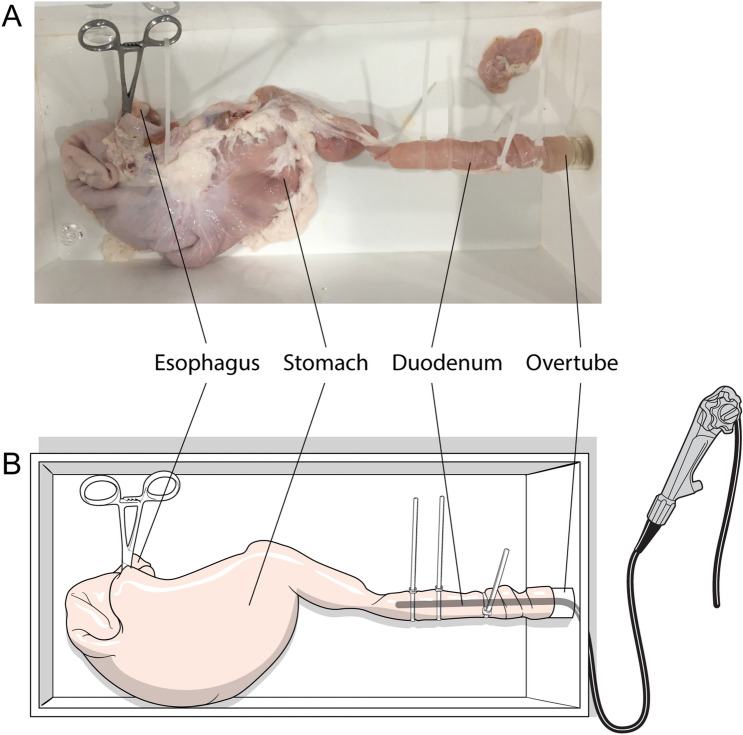
Experimental pig model (**A**) and the method of endoscope insertion for evaluating damage and perforation (**B**). The tissue was surgically removed from the pig and prepared. The esophagus was closed up by Kelly forceps and a tube measuring 18 mm in diameter was inserted through the small intestine into gastric cavity.

The MRI system used in this experiment was the Siemens 3T Skyra model (syngo MR D13). Detailed parameters for the endoclips are shown in Table 1 and Fig. 1. Porcine tissue consisting of the stomach and small intestines was donated by Olympus (Korea). The esophagus was closed using Kelly forceps during endoscopy (Fig. 2). We removed the Kelly forceps and kept the porcine tissue in a rectangular, transparent plastic container (61.2 cm × 40.8 cm × 34.5 cm) during MRI. We obtained imaging sequences of each model for 45 min since this is the normal exposure timeframe for patients.

**Table 1:** The characteristics and measured masses of separated endoclip parts.

Model name	Model no	Manufacturer	Arm length (mm)	Clip configuration	Metallic Part 1 (mg)	Metallic Part 2 (mg)	Non-metallic part (mg)
EZ clip	HX-610-135L	Olympus Medical Systems Corp	9	2 arms	32.8	17.3	12.2

### Assessment of tissue damage, perforation assessment and detachment

Two Olympus EZ clips (HX-610-135L) and one QuickClip Pro (HZ-202LR) clip were placed in the stomach and another three clips (two Olympus EZ and one QuickClip Pro) in the lumen of the small intestine. After the endoclip-tissue complex was exposed to the 3T MRI magnetic field for 45 min and imaging sequences obtained, endoscopy was performed. The endoscope was inserted through the small intestine towards the stomach to identify tissue damage, perforation, or endoclip detachment.

### Detachment force measurement

**Figure 3:**
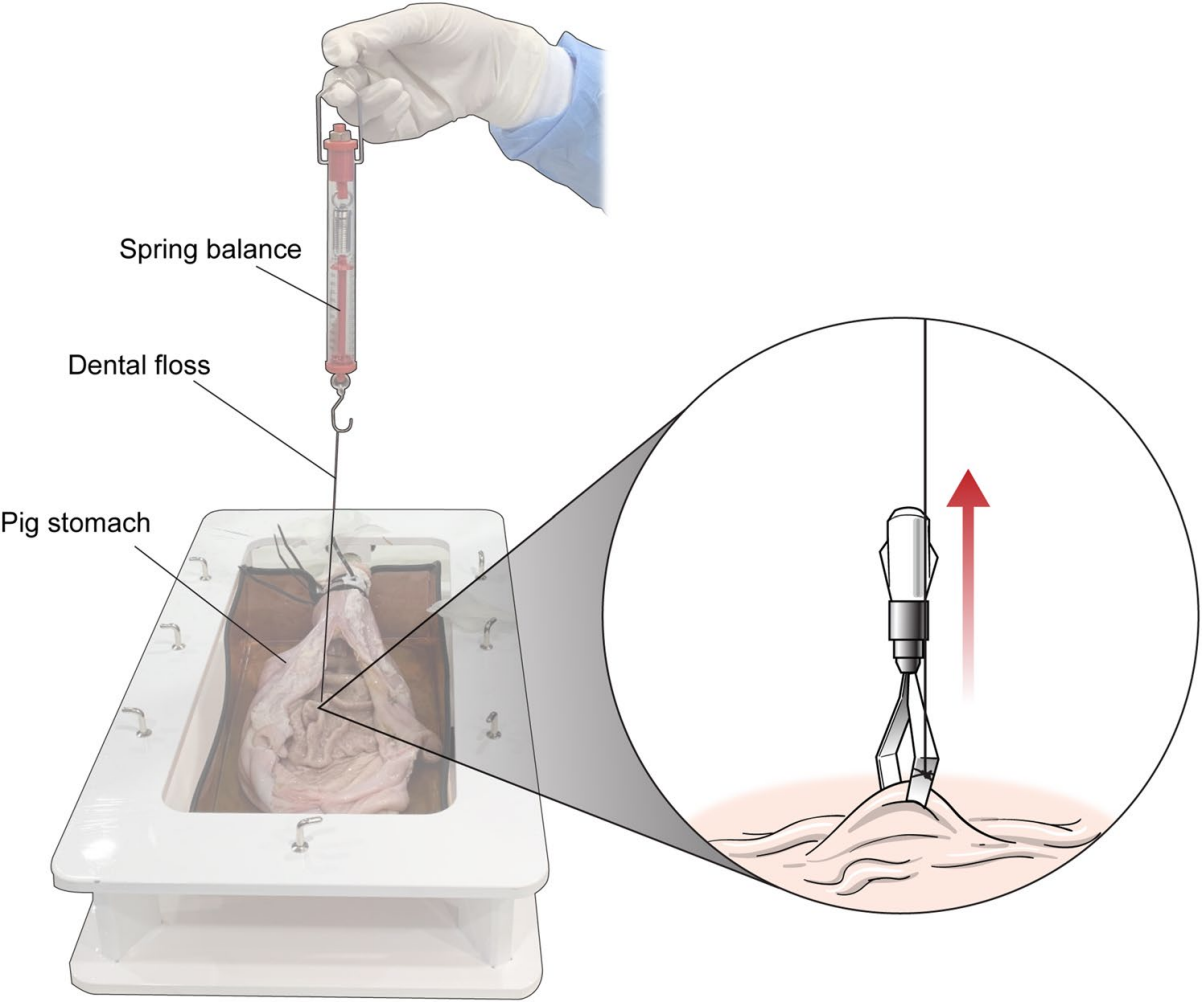
Application of the endoclip and assessment of the endoclip detachment force with a spring balance. The endoclip was placed on the surface of the mucosa and attached to a spring balance with dental floss. The spring balance was gently pulled, perpendicular to the endoclip placement, until the clip was completely detached.

To determine how much force is required to detach the endoclips, one Olympus EZ clip was attached to the stomach and another to the small intestine. The clips were connected to a spring balance using dental floss (Fig. 3). The QuickClip Pro clip was not included in this part of the experiment since this type of endoclip has been shown to be compatible with MRI. With the porcine tissue placed in a transparent plastic container, the spring balance was gently pulled, in a direction perpendicular to the mucosal surface until the endoclip was detached. The magnitude of force required to detach the clip was measured immediately when the clip separated from the mucosa. The experiment was repeated twice. Measurements were obtained for endoclips placed normally and for those pushed further into the tissue, mimicking deeper attachment.

### Measurement of magnetic translation forces acting on the endoclips

**Figure 4:**
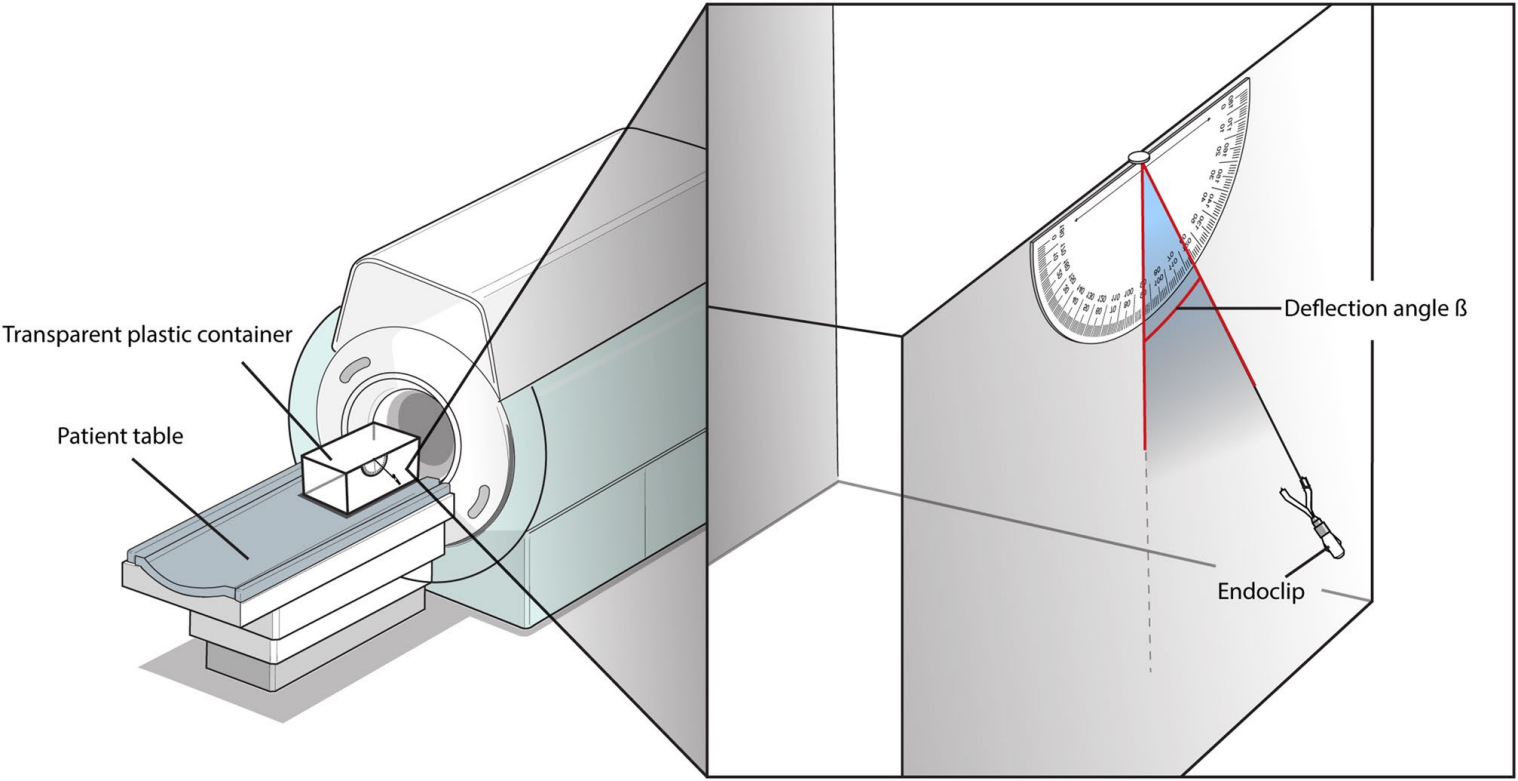
Measurement of translational forces acting on the endoclip under 3 T MRI. The endoclip was set up in a transparent plastic container and placed on the MRI patient table. The deflection angle, ß, was measured according to ASTM guidelines.

The Olympus EZ and QuickClip Pro clips were installed on the GI tract tissue mucosa. Although QuickClip Pro clips are safe to use during MRI, their magnetic translation force was measured for confirmation. After confirming deflection of each endoclip, magnetic translation forces were measured. The metallic and non-metallic parts of the Olympus EZ clip were separated and only the metallic parts were used to measure translational forces caused by paramagnetism under a 3T MRI magnetic field. As recommended by the American Society for Testing and Materials (ASTM) standards for measuring the magnetic force of a medical material, a protractor was placed inside the plastic container with a thread connected to the endoclip, in order to measure the deflection angle^3^. The container was then placed inside a 3T magnetic bore. For any clips with a deflection angle greater than 60° during the initial measurement, the translational force (F_z_) was estimated using the angle measured after a light plastic, non-ferromagnetic weight was attached (Fig. 4) according to the formula:

$${\text{F}}_{{\text{z}}} = {\text{mg}}\,{\tan}\, \, {\ss},$$

where m is the mass of the device, g is gravity (9.81 m/sec^2^), and ß is the measured angle of deflection.

### Theoretical estimation of magnetic translation forces acting on the endoclip

For an MRI device that has a bore with a solenoid coil, the magnetic field inside the solenoid coil filled with air can be expressed as follows:

$${\text{B}}_{{\text{o}}} = \mu_{{\text{r}}} \mu_{{\text{o}}} {\text{ni}},$$

where μ_r_ is the relative permeability air (μ_r_ of air = 1.0), μ_o_ is the permeability constant (calculated to be 4π × 10^–7^ T m/A), n is the number of winding layers and i is the electrical current of the coil.

If a uniform magnetic field is present inside the bore, there should be no magnetic force acting on the clip located inside the bore. Although a non-uniform magnetic field is present inside the solenoid coil, the extent of non-uniformity is presumably smaller than that outside the solenoid coil. The most critical factor is the maximum magnetic force acting on the clip. Since the clip placed outside the coil is subjected to a non-uniform magnetic field, the maximum magnetic force acts on it. The magnitude of the magnetic force is mainly determined by the distance (d) between the clip and the solenoid inlet along the central axis of the bore. The magnetic field, *B*(d) outside the solenoid coil can be expressed by the Biot-Savart law as follows^4^:

$${\varvec{B}}\left( {\mathbf{d}} \right) = \frac{{{\mathbf{B}}_{0 } }}{2}\left[ {\frac{{{\mathbf{d}} + {\mathbf{L}}}}{{\sqrt {\left( {{\mathbf{d}} + {\mathbf{L}}} \right)^{2} + {\mathbf{R}}^{2} } }} - \frac{{\mathbf{d}}}{{\sqrt {{\mathbf{d}}^{2} + {\mathbf{R}}^{2} } }}} \right],$$

where L is the coil length and R is the radius of the coil.

The magnetic force (F_m_) acting on a paramagnetic material placed in a space where the magnetic field is not uniform can be calculated as follows:

$${\mathbf{F}}_{{\mathbf{m}}} = \frac{{{\varvec{\upchi}}}}{{{\mathbf{\mu o}}}}{\mathbf{VB}}\left( {\mathbf{d}} \right)\frac{{{\mathbf{dB}}}}{{{\mathbf{dx}}}},$$

where χ is the magnetic susceptibility and V is the volume of the clip.

## Results

### Assessment of tissue damage and perforation

Following exposure to a magnetic field for 45 min during MRI, no noticeable tissue damage or perforation was observed during endoscopy of both the stomach and the small intestine.

### Detachment assessment

After MRI lasting 45 min, endoscopic findings showed that the clips remained completely attached.

### Detachment force measurement

Detachment force was only determined for the Olympus EZ clips because QuickClip Pro clips do not have magnetic properties and are compatible with MRI. Using a spring balance for measurement, force required to detach the Olympus EZ clip from the stomach mucosa was 0.9 N when endoclips were placed normally and 2.5 N when endoclips were attached deeply (Table 2). The detachment force from the small intestine mucosa was 1.2 N when endoclips were placed normally and 3.0 N when endoclips were attached deeply.

**Table 2:** Measured force of detachment for an endoclip.

Model	EZ clip (HX-610-135L) (N)
Stomach mucosa	0.9–2.5
Small intestine	1.2–3.0

### Measurement of translational forces and theoretical estimation of magnetic forces acting on the endoclips

The maximum deflection angle according to distance from the MRI inlet was measured with a protractor to identify the maximized magnetic force acting on the endoclips (Fig. 4). The deflection angle of QuickClip Pro clips, which are designed to be compatible with MRI, could not be measured because they do not have magnetic properties. The modified deflection angle of the Olympus EZ clip was 34.0^◦^ after the addition of a non-ferromagnetic weight. Using the measured modified deflection angle, the magnetic force acting on the metallic part was 3.18 × 10^–3^ N (Table 3).

**Table 3:** Calculated metallic part volume from density and translational force from deflection angle.

Model name/ Model no	Density (g/cm^3^)	Metallic part total mass (mg)	Metallic part volume (m^3^)	Measured deflection angle ß [◦]	Translational force Fz (N)
EZ clip (HX-610-135L)	7.93	50.1	6.31 × 10^–9^	34.0	3.18 × 10^–3^

## Discussion

There is currently a lack of established guidelines that summarizes the effect of MRI magnetic fields on endoclips. According to a Canadian policy survey reported in 2017, the in vivo behavior of endoclips and the risks associated with MRI-exposed endoclips have yet to be fully determined despite preliminary evidence suggesting that all endoscopic clips might not be compatible with MRI^5^.

Although Olympus EZ clips are used worldwide, some countries have prohibited MRI in patients having these clips. They recommend removal or natural excretion of the endoclips prior to MRI. However, removing these endoclips through endoscopy may cause complications such as bleeding and lead to extra costs. For natural excretion, a previous study by Jensen et al. reported that the median clip retention time was 2 weeks for the QuickClip and 4 weeks for the Resolution clip^6^. The information provided by the manufacturer, Olympus (Korea), states that the suggested average retention period of the endoclip in the gastrointestinal lumen is 9.4 days. However, it is possible for a clip to remain in the gastrointestinal tract for an extended period of time, with clips reported to have remained in the human gut for up to 33 weeks after placement^7^. These longer retention times also raise concerns over the timing of MRI. Olympus EZ clips are considered MRI incompatible although there have been no reports of complications in patients.

We conducted this study to identify tissue damage or perforation by the endoclips when exposed to MRI, and to compare the detachment force with the magnetic force acting on the endoclips when exposed to magnetic fields. In this experiment, two Olympus EZ clips and one QuickClip Pro clip were inserted into the bowel lumen and placed under a 3T magnetic field for 45 min. The QuickClip Pro clip, which is compatible with MRI, caused no tissue damage or perforation. The Olympus EZ clip, though having ferromagnetic properties, also caused no tissue damage or perforation. These results were expected because the force required to generate bowel perforation by the free end of the clip was assumed to be much greater than the force required to detach the clip. Therefore, it is unlikely that complications will occur due to the magnetic field generated during MRI if an endoclip remains in the GI tract for a certain period of time. Olympus EZ clips detached from the mucosa of the stomach and small intestine at a force of 0.9 N and 1.2 N, respectively. The QuickClip Pro clip and the Olympus EZ clip did not detach from the bowel mucosa during MRI. This finding suggests that the magnetic force acting on the clip was less than the force required to detach the clip from the mucosa. In an additional experiment designed according to ASTM recommendations, a force of 3.18 × 10^–3^ N was measured. This force is much smaller than that required to separate the clip from the mucosa (0.9 N).

MRI compatibility data for some endoclips are available online (https://mrisafety.com). While most studies addressing compatibility of clips and MRI are based on 1.5T MRI^1,5^, the magnetic force of an endoclip under a magnetic field depends on the intensity of the magnetic field. For example, the magnitude of force almost doubled for a 3T magnetic field compared to a 1.5T field^8^. Therefore, an endoclip that is considered safe for 1.5T MRI might be unsafe for a 3T or 9T MRI. In other words, the decision on whether to perform MRI is specific to a given situation. It is possible that a magnetically induced displacement force would not harm a patient. For instance, the maximum force acting on the bowel wall during colonoscopy in an animal model was found to be 12.73 N (average force 0.284 N)^9^, which is much higher than the measured translational magnetic force (3.18 × 10^–3^ N). In the human body, the MRI compatibility of a given device depends on its anatomical location. For cerebral vascular clips, paramagnetism itself can result in very dangerous complications^10^. However, ferromagnetism is not a critical issue for partial dentures fixed to teeth^11^. For endoclips, it is possible that detachment might not occur at a force of 0.9 N or less. Moreover, even separated clips with free ends could be considered compatible with MRI because a greater force is required to cause complications such as perforation. Because the retention period of an endoclip could extend to 33 weeks^7^, and delaying essential MRI evaluations could have serious consequences for patients with comorbidities such as acute stroke or malignancy, it is not advisable to unconditionally prohibit MRI for patients with paramagnetic clips.

This study has some limitations. Firstly, since this experiment was performed using normal gastric and small intestine tissues obtained from pigs within 12 h of death, the strength and tolerance might not be equivalent to those of human tissues. There is variability in strength and tolerance of different tissue types (e.g. esophagus or colon) and in the presence of diseases like ulcers. Secondly, we performed a small number of experiments using endoclips provided by a single manufacturer. The characteristics of endoclips such as length, configuration and metallic properties may affect the strength and anchoring tolerance. Further studies on animal models are required including different organs, and using a wider variety of clips.

## Conclusion

Our study demonstrated that although Olympus EZ clips have ferromagnetic properties, tissue damage, perforation or detachment during 3T MRI was not observed. Magnetic forces acting on the endoclips were measured based on the data sheet of the endoclips and features of the MRI machine. Endoclips in the GI tract may be compatible and safe to use during 3T MRI.
