# Author Correction: Compatibility of endoclips in the gastrointestinal tract with magnetic resonance imaging

**DOI:** 10.1038/s41598-021-85467-0

**Published:** 2021-03-11

**Authors:** Dong Yeol Shin, Sumi Park, Ain Kim, Eung-Sam Kim, Han Ho Jeon

**Affiliations:** 1 Division of Gastroenterology, Department of Internal Medicine, National Health Insurance Service Ilsan Hospital, 100 Ilsan-ro Ilsan-donggu, Goyang-si, Goyang, 10444 Korea; 2 Department of Radiology, National Health Insurance Service Ilsan Hospital, Goyang, Korea; 3 Department of Human Biology, University of Toronto, Toronto, ON Canada; 4 Department of Biological Sciences and Research Center of Ecomimetics, Chonnam National University, Gwangju, Korea

Correction to: *Scientific Reports* https://doi.org/10.1038/s41598-020-73726-5, published online 06 October 2020

The original version of this Article contained an error in the Abstract.

“The translational magnetic force acting on the endoclips was 3.18 × 10^–3^ N. Ex vivo experiments showed that the magnetic field generated by 3 MRI did not cause tissue damage or perforation and did not detach the endoclips. Olympus EZ clips and QuickClip Pro clips in the GI tract appear to be safe during 3T MRI”.

now reads:

“The translational magnetic force acting on the endoclips was 3.18 × 10^–3^ N. Ex vivo experiments showed that the magnetic field generated by 3T MRI did not cause tissue damage or perforation and did not detach the endoclips. Olympus EZ clips and QuickClip Pro clips in the GI tract appear to be safe during 3T MRI.”

This error has now been corrected in the PDF and HTML versions of the Article.

